# On the Phase Separation in *n*-Type Thermoelectric Half-Heusler Materials

**DOI:** 10.3390/ma11040649

**Published:** 2018-04-23

**Authors:** Michael Schwall, Benjamin Balke

**Affiliations:** Institut für Anorganische und Analytische Chemie, Johannes Gutenberg-Universität, 55099 Mainz, Germany; balke@uni-mainz.de

**Keywords:** Heusler compounds, phase separation, thermoelectrics

## Abstract

Half-Heusler compounds have been in focus as potential materials for thermoelectric energy conversion in the mid-temperature range, e.g., as in automotive or industrial waste heat recovery, for more than ten years now. Because of their mechanical and thermal stability, these compounds are advantageous for common thermoelectric materials such as Bi${}_{2}$Te${}_{3}$, SiGe, clathrates or filled skutterudites. A further advantage lies in the tunability of Heusler compounds, allowing one to avoid expensive and toxic elements. Half-Heusler compounds usually exhibit a high electrical conductivity σ, resulting in high power factors. The main drawback of half-Heusler compounds is their high lattice thermal conductivity. Here, we present a detailed study of the phase separation in an *n*-type Heusler materials system, showing that the Ti${}_{x}$Zr${}_{y}$Hf${}_{z}$NiSn system is not a solid solution. We also show that this phase separation is key to the thermoelectric high efficiency of *n*-type Heusler materials. These results strongly underline the importance of phase separation as a powerful tool for designing highly efficient materials for thermoelectric applications that fulfill the industrial demands of a thermoelectric converter.

## 1. Introduction

The search for alternative energy technologies has accelerated in recent years as climate change has become more noticeable and the use of nuclear energy introduces political controversy for many countries. The quest for sustainable energy sources has piqued interest in different research fields to find new energy conversion techniques to satisfy the world’s rising demand for energy. In 2016, the Lawrence Livermore National Laboratory suggested that more than 66% of the energy that flows through our economy is ultimately wasted [1]. Most of the energy is dissipated as waste heat, and only 25% is used as mechanical power [2,3]. Some of this thermal energy could be converted directly into electrical energy by a thermoelectric generator (TEG) [4,5]. Presently, thermoelectric (TE) devices are actively considered as a clean energy source for waste heat recovery in automobiles, since they operate silently and do not have any moving parts or environmentally-harmful fluids. The captured energy can be used for a vehicle’s electrical components such as air conditioning, lights and windows without additional engine load [6]. This in turn can make a crucial contribution to an improvement in fuel economy and thus a reduction of CO${}_{2}$ emissions. Beside waste heat recovery, TE devices have been investigated for their use in TE-solar hybrid systems [4], TE-refrigeration [7] and as radioisotope TEGs for the deep-space applications of NASAs Voyager and Cassini missions [8].

Half-Heusler (HH) compounds are some of the most promising candidates for thermoelectric materials for automotive and industrial waste heat recovery applications. A general challenge in improving HH compounds for thermoelectric applications is the comparatively high thermal conductivity of the order of 10 WK${}^{-1}$m${}^{-1}$, while other classes of TE materials like bismuth tellurides, lead tellurides and skutterudites reach values below 2 WK${}^{-1}$m${}^{-1}$. In the late 1990s a common approach to reduce the thermal conductivity was to increase the phonon scattering. Hohl et al. reduced the thermal conductivity by a factor of three for different temperatures by introducing disorder on the *X*-sites of $X_{0.5}X_{0.5}^{\prime }$NiSn ($X,X^{\prime }$ = Ti, Zr, Hf) [9,10]. Substitution of tin by antimony increases both the thermal and electrical conductivities [11]. Substitution at the Ni position also decreases the thermal conductivity [12]. The composition Zr${}_{0.5}$Hf${}_{0.5}$Ni${}_{0.8}$Pd${}_{0.2}$Sn${}_{0.99}$Sb${}_{0.01}$ possesses a figure of merit ZT = 0.7 at 527 ${}^{\circ }$C [13].

For a long time, the TE applicability of HH compounds was limited by their high thermal conductivities κ > 7–10 WK${}^{-1}$m${}^{-1}$ [14]. The introduction of mass disorder due to site substitution (alloying) [15,16] and nanostructures [17,18] is an effective way to produce additional phonon scattering and with it to decrease the thermal conductivity. Thus, the substitution of non- and isoelectronic elements leads to a drastic decline in the thermal conductivity (κ< 4 WK${}^{-1}$m${}^{-1}$) of HH materials [19]. The resulting higher disorder due to higher mass and strain fluctuations and an intrinsic phase separation in multi-component HH materials are responsible for the strong reduction in κ. Another approach is to implement a nano- or micro-structure in the thermoelectric material. This can be achieved by phase separation, composite materials, pulverization with additional spark plasma sintering or by a complex lattice structure [20,21]. The experimental efforts of site substitution in HH compounds significantly improved their TE performance. Hereby, ZT values of 1.5 at 427 ${}^{\circ }$C in the *n*-type Zr${}_{0.25}$Hf${}_{0.25}$Ti${}_{0.5}$NiSn${}_{0.998}$Sb${}_{0.002}$ [19,22] and ZT ≈ 1 in the *p*-type Ti${}_{0.12}$Zr${}_{0.44}$Hf${}_{0.44}$CoSn${}_{0.8}$Sb${}_{0.2}$ material [16] were attained.

Our first contribution to the field of phase separation was the investigation of the the phase separation of the solid solution CoTi${}_{\left(1-x\right)}$Mn${}_{x}$Sb into the two Heusler compounds CoTiSb and CoMnSb [21]. EDX measurements on the two-phase material revealed the presence of size and shape tunable CoTiSb regions in a CoMnSb matrix. We demonstrated that the formed phase and grain boundaries have a considerable influence on the phonon scattering processes, which leads to a reduction of the thermal conductivity by a factor of three compared to the single-phase compound CoTiSb. In contrast to the decrease of the thermal conductivity by grinding and hot pressing, there is no need for separate processing steps after the synthesis of the compounds in this approach. After this first promising results, we started to try to understand the phase separation in the Heusler compound in more detail. We investigated the series of the quaternary Heusler compound Co${}_{2}$Mn${}_{\left(1-x\right)}$Ti${}_{x}$Sn with (*x* = 0, 0.2, 0.4, 0.5, 0.6, 0.8, 1) and observed that this series also shows a phase separation [20]. The quaternary compounds have a dendritic microstructure, which was formed during the solidification process as a result of a phase separation into a Co${}_{2}$MnSn-enriched and a Co${}_{2}$TiSn-enriched Heusler phase.

Of course, there are other techniques to improve the thermoelectric performance in these Heusler materials. Chen and co-workers achieved via an improved structural order through proper annealing a ZT of around 1.2 in Hf${}_{0.6}$Zr${}_{0.4}$NiSn${}_{0.995}$Sb${}_{0.005}$ [23]. The same group studied the role of prospective resonant dopants and showed that doping of vanadium could introduce resonant states. Using this approach, they obtained enhanced Seebeck coefficients due to the presence of V resonant states and a reduced thermal conductivity resulting in a ZT of around 1.3 in the material (Hf${}_{0.6}$Zr${}_{0.4}$)${}_{0.99}$V${}_{0.01}$NiSn${}_{0.995}$Sb${}_{0.005}$ [24]. Fu et al. reported a high ZT of around 1.5 at 1200 K for the *p*-type FeNbSb heavy-band half-Heusler alloys. The high content of the heavier Hf dopant simultaneously optimizes the electrical power factor and suppresses thermal conductivity. Both the enhanced point-defect and electron–phonon scatterings contribute to a significant reduction in the lattice thermal conductivity [25].

As already mentioned above, Toshiba (Japan) reported a maximum ZT of 1.5 for Zr${}_{0.25}$Hf${}_{0.25}$Ti${}_{0.5}$NiSn${}_{0.998}$Sb${}_{0.002}$ at 427 ${}^{\circ }$C [19,22]. These high ZT values were never reproduced by any other group since the original publication in 2005. In 2013, we were able to show that the high figure of merit values reported by Shutoh and Sakurada [19,22] could almost be reproduced [26]. The origin of the exceptional low thermal conductivity is the phase decomposition, which does not influence the electrical conductivity significantly because of semi-coherent interfaces existing between the three co-existing Heusler phases. The deviations in the electronic properties in comparison to the data reported by Shutoh and Sakurada are probably due to different measurement setups and a different synthesis method. Additionally, we showed that the Heusler compounds are tunable by doping with several elements, which allows one to design these materials for a particular application. The shown reproducibility of the high figure of merit of the Heusler compounds makes these materials one of the most interesting material classes for high temperature thermoelectric applications.

## 2. Results

### 2.1. Understanding the Phase Separation in the *n*-Type HH Materials

The goal of this very detailed and systematic investigation of the phase separation in the *n*-type HH materials was to understand the phase separation in these materials. When we started this investigation, the best four reported ZT’s for Heusler compounds in the field of thermoelectricity were derived from the Ti${}_{x}$Zr${}_{y}$Hf${}_{z}$NiSn system. The idea of this work was to investigate why this Ti${}_{x}$Zr${}_{y}$Hf${}_{z}$NiSn system exhibits such promising properties and what could be the possible reasons for this. For better readability, the weighted samples are named as Ti${}_{x}$Zr${}_{y}$Hf${}_{z}$NiSn and the detected phases as Ti${}_{x}$Zr${}_{y}$Hf${}_{z}$NiSn (roman numerals).

**Figure 1:**
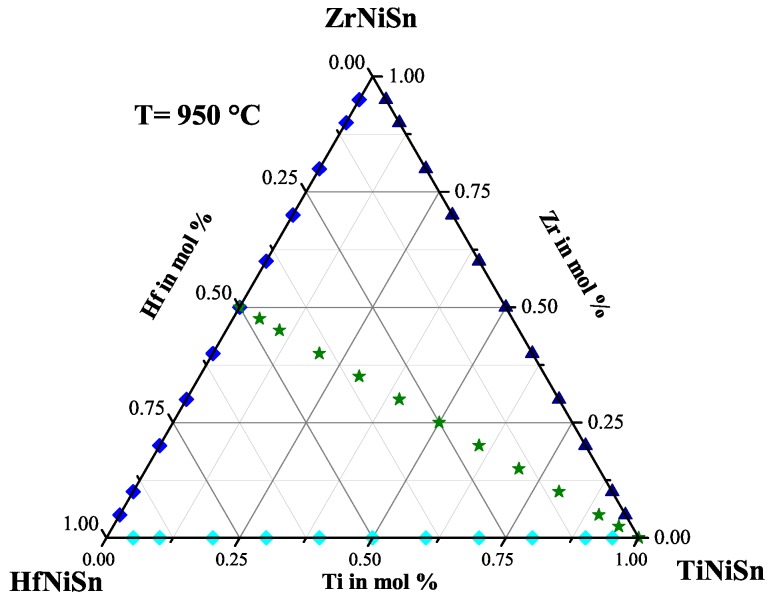
The stoichiometry of the produced samples is shown in the quasi-ternary phase diagram.

For this systematic investigation of the phase separation, we produced a large number of different samples. All samples were prepared by our conventional preparation method. The constituting elements were stoichiometrically weighed, then arc melted three times with our home-made system. After this, the samples were crushed and remelted, to ensure the homogeneity of the sample. This procedure was followed by an additional heat treatment for seven days at 950 ${}^{\circ }C$ under argon atmosphere in quartz ampules. Figure 1 shows the quasi-ternary phase diagram with the pure phases TiNiSn, ZrNiSn and HfNiSn in the corners. The scatters show the stoichiometry of the produced samples within this triangle. To reduce the number of parameters for this investigation, we first produced the samples on the sides of this triangle, and later on, we produced the samples in the inner part of the triangle with the stoichiometry Ti${}_{\left(1-x\right)}$(Zr${}_{0.5}$Hf${}_{0.5}$)${}_{x}$NiSn. In the following, the results of this investigation are presented.

### 2.2. The Zr${}_{\left(1-x\right)}$Hf${}_{x}$NiSn Series

**Figure 2:**
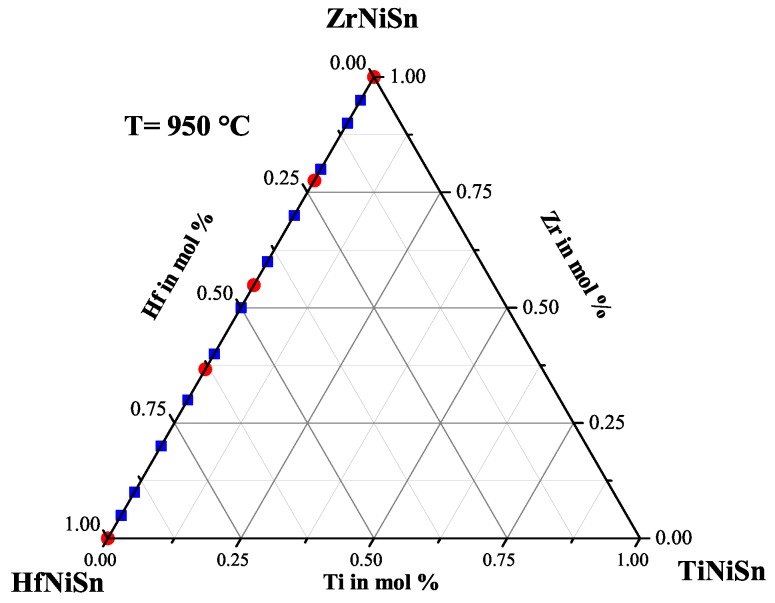
The Zr${}_{\left(1-x\right)}$Hf${}_{x}$NiSn series with the detected phases indicated in the Gibbs triangle.

To further reduce the number of parameters for the investigation, Ti was ignored, and the Zr${}_{\left(1-x\right)}$Hf${}_{x}$NiSn series was produced. The lattice parameters a and the phase compositions are shown in Table 1. The obtained lattice parameters, achieved by multiple phase fit, indicate a dependence on the Hf concentration, but through comparison of the detected phases by EDX, just five different C1${}_{b}$ phases were found. Besides the parent phases ZrNiSn and HfNiSn, the phases with average compositions of Zr${}_{0.78}$Hf${}_{0.22}$NiSn(XII), Zr${}_{0.55}$Hf${}_{0.45}$NiSn(XIII) and Zr${}_{0.36}$Hf${}_{0.64}$NiSn(XIV) are detected (see Table 2). This indicates that the Zr${}_{\left(1-x\right)}$Hf${}_{x}$NiSn series is not a solid solution and that just a few compositions of Zr${}_{\left(1-x\right)}$Hf${}_{x}$NiSn are stable at a temperature of 950 ${}^{\circ }C$. In Figure 2, the produced compositions (blue) and the detected phases (red) are indicated in the Gibbs triangle for the Ti${}_{x}$Zr${}_{y}$Hf${}_{z}$NiSn system. It is remarkable that it was not possible to resolve the coexistence of two Heusler C1${}_{b}$ phases in one compound by standard XRD measurements in the laboratory.

**Figure 3:**
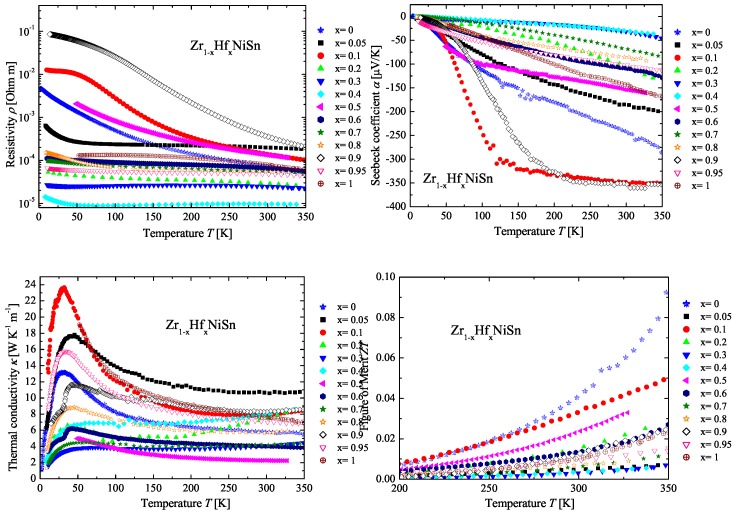
Electrical resistivity (**top left**), Seebeck coefficient (**top right**), thermal conductivity (**bottom left**) and figure of merit (**bottom right**) of the Zr${}_{\left(1-x\right)}$Hf${}_{x}$NiSn series in comparison to the parent compounds.

In Figure 3, the measured physical properties of the Zr${}_{\left(1-x\right)}$Hf${}_{x}$NiSn series are compared with the parents ZrNiSn and HfNiSn. All obtained values of the Seebeck coefficient (see Figure 3 (top right)) for the Zr${}_{\left(1-x\right)}$Hf${}_{x}$NiSn series show a negative value, which indicates *n*-type conduction. The first impression of the different developments is that there is no dependence among the measurements, but if the values of the Seebeck coefficient are set in relation to the detected stable phases (see Table 1 or Figure 2), a clear trend is recognizable. All samples that consist of only one phase, meaning that they exhibit almost a stable composition, have high values for the Seebeck coefficient (ZrNiSn, Zr${}_{0.1}$Hf${}_{0.9}$NiSn, Zr${}_{0.5}$Hf${}_{0.5}$NiSn, Zr${}_{0.9}$Hf${}_{0.1}$NiSn, Zr${}_{0.95}$Hf${}_{0.05}$NiSn, HfNiSn). This is not unexpected because for high values of the Seebeck coefficient, a good crystalline structure is needed, meaning that no shortcut by impurity phases exists. Structural stress, disorder or impurities increase the number of states next to the conduction band and decrease the gap to the conduction band by broadening of the localized states. This decreases the values of the Seebeck coefficient and decreases the resistivity. In Figure 3 (top left), this can be seen in the resistivity values. The resistivity of the different Zr${}_{\left(1-x\right)}$Hf${}_{x}$NiSn samples follows the same trend as the values of the Seebeck coefficient. Except sample Zr${}_{0.5}$Hf${}_{0.5}$NiSn, all samples with high values of the Seebeck coefficient exhibit a high lattice thermal conductivity due to the good crystallinity and the low impurities. Although the samples with more phases exhibit low values for the resistivity, meaning that the electronic contribution to the thermal conductivity $\kappa_{e}$ is high, the thermal conductivity κ is low (see Figure 3 (bottom left)). This is the effect of the phase separation, which causes additional phonon scattering at the grain and phase boundaries. The lowest value is achieved by the stable phase Zr${}_{0.5}$Hf${}_{0.5}$NiSn with a value of κ = 2.2 WK${}^{-1}$m${}^{-1}$. The sample with a phase decomposition, Zr${}_{0.7}$Hf${}_{0.3}$NiSn, exhibits a low value for the thermal conductivity, as well. The samples with a phase segregation, meaning with at least two phases with different crystal structures coexisting, also have low values for the thermal conductivity, and they show the typical behavior of a compound with a complex unit cell in a quasicrystal or amorphous metal [27,28].

### 2.3. The Ti${}_{\left(1-x\right)}$Zr${}_{x}$NiSn Series

**Figure 4:**
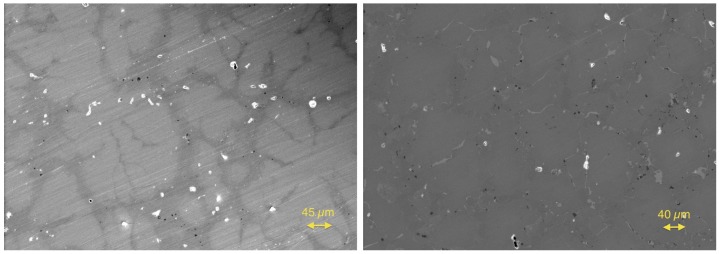
Scanning electron microscope images of Ti${}_{0.4}$Zr${}_{0.6}$NiSn (**left**) and Ti${}_{0.6}$Zr${}_{0.4}$NiSn (**right**).

After the investigation of the Zr/Hf system, the Hf was ignored, and the Ti${}_{\left(1-x\right)}$Zr${}_{x}$NiSn series was investigated to prove that this series is not a solid solution either. The lattice parameters a and the phase compositions are listed in Table 3. A slight trend is recognizable in the obtained lattice parameters showing a dependence of the Zr concentration. The comparison of the detected phases again leads to just five different C1${}_{b}$ phases. In addition to the parent phases ZrNiSn and TiNiSn, the phases Ti${}_{0.80}$Zr${}_{0.19}$NiSn(IX), Ti${}_{0.66}$Zr${}_{0.34}$NiSn(X) and Ti${}_{0.29}$Zr${}_{0.71}$NiSn(XI) were found (see Table 4). The analogy to the detected phases of the Zr${}_{\left(1-x\right)}$Hf${}_{x}$NiSn series is remarkable and shows that the series are chemically related. This result is not unexpected because the properties of Ti, Zr and Hf are related. Two examples of SEM images of a phase-separated and an almost clean sample are shown in Figure 4.

**Figure 5:**
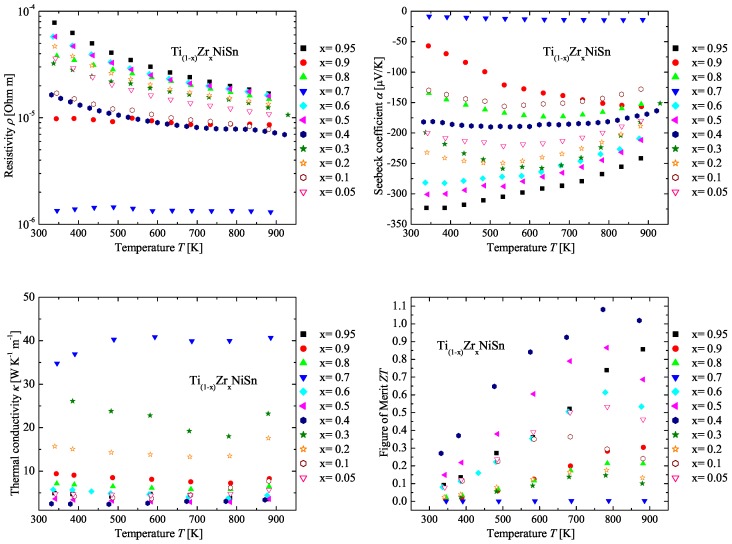
Electrical resistivity (**top left**), Seebeck coefficient (**top right**), thermal conductivity (**bottom left**) and figure of merit (**bottom right**) of the Ti${}_{\left(1-x\right)}$Zr${}_{x}$NiSn series in comparison to the parent compounds.

**Figure 6:**
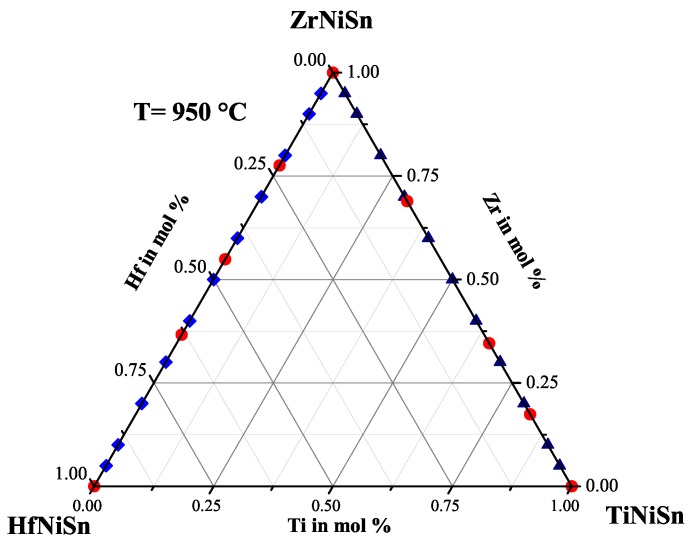
The Ti${}_{\left(1-x\right)}$Zr${}_{x}$NiSn series with the detected phases indicated in the Gibbs triangle.

In Figure 5, the measured physical properties of the Ti${}_{\left(1-x\right)}$Zr${}_{x}$NiSn series are compared. All samples of the Ti${}_{\left(1-x\right)}$Zr${}_{x}$NiSn series exhibit *n*-type conduction indicated by the negative values of the Seebeck coefficient (see Figure 5 (top right)). Again, the values of the Seebeck coefficient of the Ti${}_{\left(1-x\right)}$Zr${}_{x}$NiSn series seem not to depend on the composition. High values of the Seebeck coefficient are obtained for Ti${}_{0.05}$Zr${}_{0.95}$NiSn, Ti${}_{0.3}$Zr${}_{0.7}$NiSn, Ti${}_{0.4}$Zr${}_{0.6}$NiSn, Ti${}_{0.5}$Zr${}_{0.5}$NiSn and Ti${}_{0.8}$Zr${}_{0.2}$NiSn. In comparison to the detected stable phases (see Table 3 or Figure 6), just samples with one Heusler phase with the C1${}_{b}$ structure or a decomposition of two Heusler phases with the C1${}_{b}$ structure exhibit the high values of the Seebeck coefficient. This shows that a good crystalline structure and low impurities are needed. Furthermore, the decomposition of two clean Heusler phases with the C1${}_{b}$ structure can lead to high values of the Seebeck coefficient. The values of the resistivity support this observation (see Figure 5 (top left)) and have the same dependence as the values of the Seebeck coefficient. The Ti${}_{\left(1-x\right)}$Zr${}_{x}$NiSn samples with high values of the Seebeck coefficient show moderate values of thermal conductivity, in contrast to the results of the Zr${}_{\left(1-x\right)}$Hf${}_{x}$NiSn series. The thermal conductivity (see Figure 5 (bottom left)) of these samples is dominated by the crystallinity of the samples. This is recognizable as well in the values of the resistivity, which are high in the samples with a clean C1${}_{b}$ structure, and leads to a low contribution to the electronic thermal conductivity $\kappa_{e}$ in comparison to the other samples of the Ti${}_{\left(1-x\right)}$Zr${}_{x}$NiSn series. In Figure 5 (bottom right), the temperature dependence of the figure of merit is shown. The samples with high values of the Seebeck coefficient show high values for the figure of merit. Furthermore, the samples with moderate values of the Seebeck coefficient and a lower resistivity in comparison to the samples with a clean C1${}_{b}$ structure show high ZT values. This is due to the high impact of the resistivity on the figure of merit. In Figure 6, the produced compositions (dark blue) and the detected phases (red) are indicated in the Gibbs triangle for the Ti${}_{x}$Zr${}_{y}$Hf${}_{z}$NiSn system. These results also lead to the conclusion that the Ti${}_{\left(1-x\right)}$Zr${}_{x}$NiSn series is not a solid solution, either.

### 2.4. The Ti${}_{\left(1-x\right)}$Hf${}_{x}$NiSn Series

**Figure 7:**
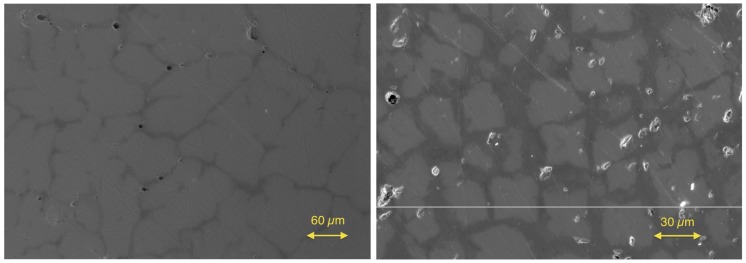
Scanning electron microscope images of Ti${}_{0.2}$Hf${}_{0.8}$NiSn (**left**) and Ti${}_{0.7}$Hf${}_{0.3}$NiSn (**right**).

**Figure 8:**
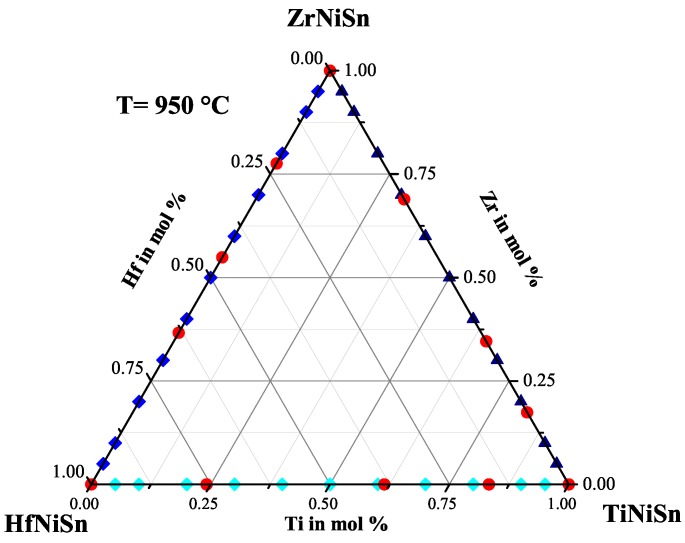
The Ti${}_{\left(1-x\right)}$Hf${}_{x}$NiSn series with the detected phases indicated in the Gibbs triangle.

The Ti${}_{\left(1-x\right)}$Hf${}_{x}$NiSn series was also investigated to verify that this series is not a solid solution either. The values of the lattice parameters a and the phase compositions are shown in Table 5. In this series, the dependence of the lattice parameters on the Hf concentration is also recognizable. Analogous to the two series above, the comparison of the detected phases lead to five different C1${}_{b}$ phases. Together with the parent phases TiNiSn and HfNiSn, the phases Ti${}_{0.83}$Hf${}_{0.17}$NiSn(VII), Ti${}_{0.64}$Hf${}_{0.36}$NiSn(VI) and Ti${}_{0.24}$Zr${}_{0.76}$NiSn(V) were found (see Table 6). Again, in comparison to the Zr${}_{\left(1-x\right)}$Hf${}_{x}$NiSn and the Ti${}_{\left(1-x\right)}$Zr${}_{x}$NiSn series, the stable Heusler C1${}_{b}$ phases found are almost the same. This result shows that the formation of stable Heusler C1${}_{b}$ phases depends on the chemical character of the constructing elements and that the atom size has almost no influence of the formation of a stable C1${}_{b}$ phase, implying that the covalent contribution to the bonding plays an important role in the formation of a stable composition. Two examples of SEM images of a phase-separated and an almost clean sample are shown in Figure 7. In Figure 8, the produced compositions (light blue) and the detected phases (red) are indicated in the Gibbs triangle for the Ti${}_{\left(1-x\right)}$Hf${}_{x}$NiSn system.

**Figure 9:**
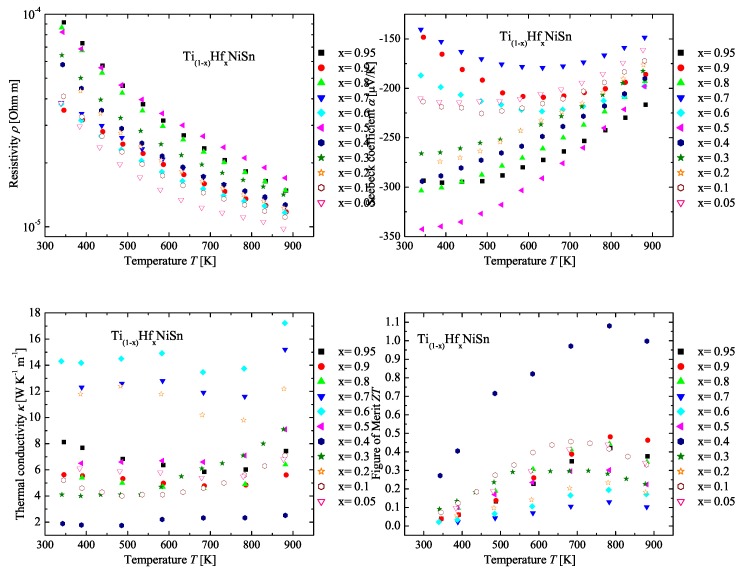
Electrical resistivity (**top left**), Seebeck coefficient (**top right**), thermal conductivity (**bottom left**) and figure of merit (**bottom right**) of the Ti${}_{\left(1-x\right)}$Hf${}_{x}$NiSn series in comparison to the parent compounds.

In Figure 9, the measured physical properties of the Ti${}_{\left(1-x\right)}$Hf${}_{x}$NiSn series are compared. All samples of the Ti${}_{\left(1-x\right)}$Hf${}_{x}$NiSn series are *n*-type semiconductors, indicated by the negative values of the Seebeck coefficient (see Figure 9 (top right)). Similar to the results in the two series above, the samples with one Heusler phase with the C1${}_{b}$ structure or a decomposition of two Heusler phases with the C1${}_{b}$ structure exhibit high values of the Seebeck coefficient. The compositions are Ti${}_{0.05}$Hf${}_{0.95}$NiSn, Ti${}_{0.2}$Hf${}_{0.8}$NiSn, Ti${}_{0.5}$Hf${}_{0.5}$NiSn, Ti${}_{0.6}$Hf${}_{0.4}$NiSn, Ti${}_{0.7}$Hf${}_{0.3}$NiSn and Ti${}_{0.8}$Hf${}_{0.2}$NiSn. Additionally, the results are in a good agreement with the stable phases (see Table 5 or Figure 8). Furthermore, the values of the resistivity follow the same trend as the values of the Seebeck coefficient (Figure 9 (top left)). The thermal conductivity of these samples seems to be dominated by the crystallinity of the samples, except the sample Ti${}_{0.6}$Hf${}_{0.4}$NiSn, which exhibits a very low conductivity. As mentioned above, the samples with a high Seebeck coefficient, high crystallinity and low impurities exhibit high thermal conductivity. The lattice thermal conductivity shows a slight increase at high temperatures, which happens due to the bipolar contribution to the conduction. In Figure 9 (bottom right), the temperature dependence of the figure of merit is shown. Similar to the Zr${}_{\left(1-x\right)}$Hf${}_{x}$NiSn series, the figure of merit is dominated by the high values of the Seebeck coefficient. This is not unexpected because the resistivity values are in the same order of magnitude, and hence, the impact on the figure of merit by the Seebeck coefficient is high.

### 2.5. The Ti${}_{\left(1-x\right)}$(Zr${}_{0.5}$Hf${}_{0.5}$)${}_{x}$NiSn Series

**Figure 10:**
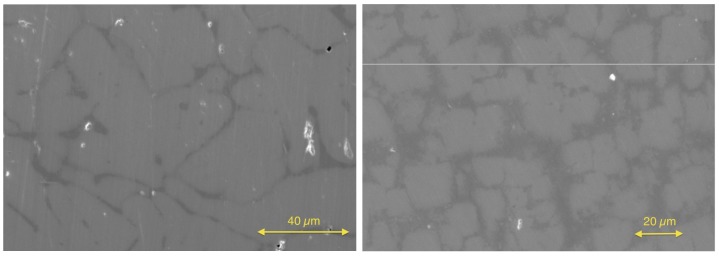
Scanning electron microscope images of Ti${}_{0.2}$(Zr${}_{0.5}$Hf${}_{0.5}$)${}_{0.8}$NiSn (**left**) and Ti${}_{0.4}$(Zr${}_{0.5}$Hf${}_{0.5}$)${}_{0.6}$NiSn (**right**).

**Figure 11:**
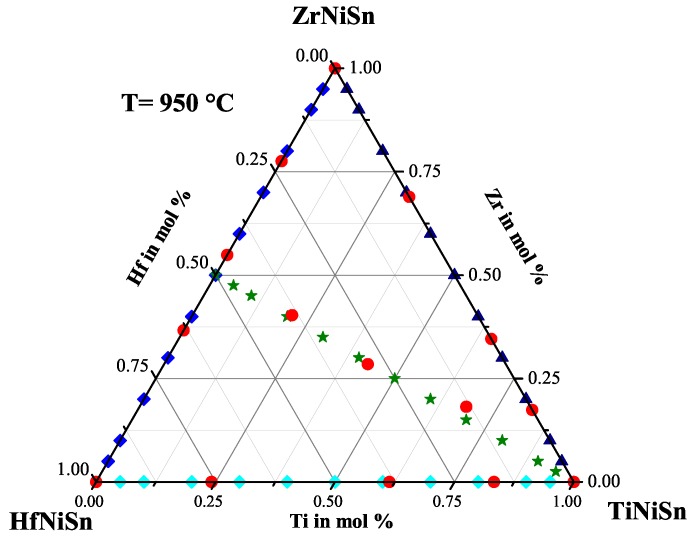
The Ti${}_{\left(1-x\right)}$(Zr${}_{0.5}$Hf${}_{0.5}$)${}_{x}$NiSn series with the detected phases indicated in the Gibbs triangle.

To combine the three series above, but to avoid the complexity of three different parameters, one parameter was fixed. With respect to that, the Ti${}_{\left(1-x\right)}$(Zr${}_{0.5}$Hf${}_{0.5}$)${}_{x}$NiSn series was investigated. Except for the samples with low Ti or (Zr${}_{0.5}$Hf${}_{0.5}$) concentrations, all samples are phase separated (see Table 7). The XRD measurements show three samples with phase separations, but the other XRD measurements of the series exhibit just a broadening of the reflections, which can indicate that a sample consists of an additional phase with a similar lattice parameter. The fitted lattice parameters a and the phase compositions are listed in Table 7. The obtained lattice parameters indicate a slight dependence on the Ti concentration, but the phases detected by EDX show that just seven different Heusler C1${}_{b}$ phases were found. Beside the parent phases (TiNiSn and Zr${}_{0.55}$Hf${}_{0.45}$NiSn(XIII)) and Ti${}_{0.82}$Zr${}_{0.18}$NiSn(IX) and Ti${}_{0.83}$Hf${}_{0.17}$NiSn(VII) found as well in the series above, only three phases were obtained: Ti${}_{0.68}$Zr${}_{0.18}$Hf${}_{0.13}$NiSn(I), Ti${}_{0.43}$Zr${}_{0.28}$Hf${}_{0.29}$NiSn(III) and Ti${}_{21}$Zr${}_{0.40}$Hf${}_{0.39}$NiSn(IV) (see Table 8). Two examples of SEM images of the phase separated samples are shown in Figure 10. The results are similar to the results of the other series. It is remarkable that stable C1${}_{b}$ phases are found from the other series above; hence, these results are consistent with the results from the other series. A slight relation seems to exist between the “binary” (Zr${}_{\left(1-x\right)}$Hf${}_{x}$NiSn, Ti${}_{\left(1-x\right)}$Zr${}_{x}$NiSn,Ti${}_{\left(1-x\right)}$Hf${}_{x}$NiSn) series and this “ternary” series (see Figure 11).

**Figure 12:**
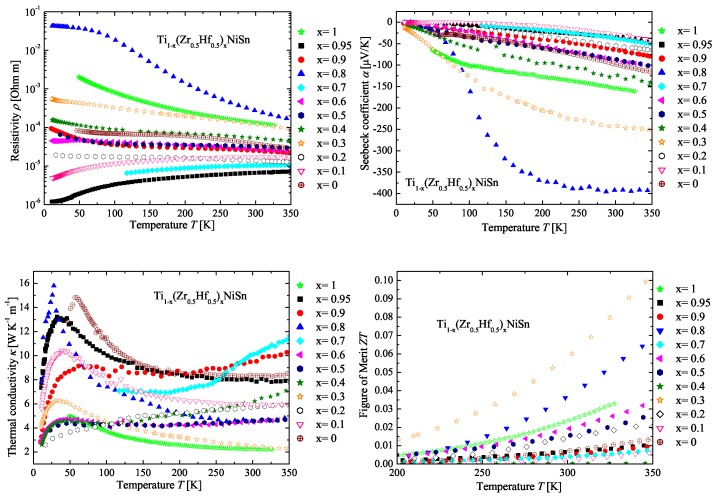
Electrical resistivity (**top left**), Seebeck coefficient (**top right**), thermal conductivity (**bottom left**) and figure of merit (**bottom right**) of the Ti${}_{\left(1-x\right)}$(Zr${}_{0.5}$Hf${}_{0.5}$)${}_{x}$NiSn series in comparison to the parent compounds.

In Figure 12, the measured physical properties of the Ti${}_{\left(1-x\right)}$(Zr${}_{0.5}$Hf${}_{0.5}$)${}_{x}$NiSn series are compared to the parents TiNiSn and Zr${}_{0.5}$Hf${}_{0.5}$NiSn. All measured values of the Seebeck coefficient (see Figure 12 (top right)) for the Ti${}_{\left(1-x\right)}$(Zr${}_{0.5}$Hf${}_{0.5}$)${}_{x}$NiSn series are negative, indicating *n*-type conduction.

Analogous to the other series, the samples, which almost possess the composition of the detected stable phases, exhibit high values of the Seebeck coefficient. It is also remarkable that samples with very similar phase compositions have the same values of the Seebeck coefficient and resistivity (Ti${}_{0.4}$(Zr${}_{0.5}$Hf${}_{0.5}$)${}_{0.6}$NiSn and Ti${}_{0.5}$(Zr${}_{0.5}$Hf${}_{0.5}$)${}_{0.5}$NiSn). This shows that the measurements are consistent and that the phase composition determines the transport properties. The resistivity values support the results of the Seebeck coefficient values (Figure 12 (top left)). In the measurements of the thermal conductivity (Figure 12 (bottom left)), the impact of the phase decomposition can be seen. All samples with a decomposition of two Heusler C1${}_{b}$ phases exhibit a low lattice thermal conductivity due to the additional scattering at the grain and phase boundaries. The samples with segregation show higher thermal conductivities, because of the mixture of Heusler phases, with low lattice thermal conductivities and binary alloys, which usually exhibit high lattice thermal conductivities in comparison to these Heusler compounds. Figure 12 (bottom right) shows the temperature dependence of the figure of merit. The values of the figure of merit are dominated by the high values of the Seebeck coefficient and the mentioned low thermal conductivity.

## 3. Discussion

**Figure 13:**
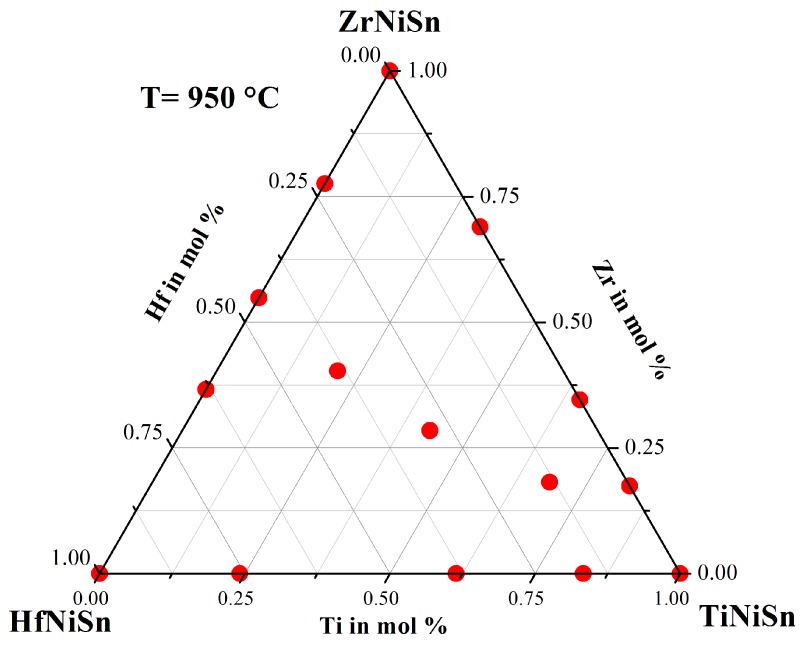
The detected stable phases at 950 ${}^{\circ }C$ indicated in the Gibbs triangle.

To prove the results from the series above, three stable Heusler phases with different Ti${}_{x}$Zr${}_{y}$Hf${}_{z}$NiSn ratios were produced and characterized. Additionally, samples with a varied Ti:Zr:Hf ratio were produced to see if the constructed Gibbs triangle works (see Figure 13). The compositions of the produced and the detected phases are shown in Table 9. All detected phases are in good agreement with the results from the series above. The constructed Gibbs triangle seems to work. After several optimization steps, the stable Heusler phases with the C1${}_{b}$ structure Ti${}_{0.21}$Zr${}_{0.40}$Hf${}_{0.39}$NiSn(IV), Ti${}_{0.43}$Zr${}_{0.28}$Hf${}_{0.29}$NiSn(III) and Ti${}_{0.68}$Zr${}_{0.17}$Hf${}_{0.15}$NiSn(I) could be synthesized as a single phase.

In summary, in the quasi-binary Zr${}_{\left(1-x\right)}$Hf${}_{x}$NiSn system, only three stable phases were determined, apart from the parent compounds. The thermal conductivity of the samples with almost one phase is, in comparison to the parent compounds, remarkably low, but the electronic properties are not adequate for thermoelectric use. This is due to the fact that the pure stable compounds were not synthesized. The sample Zr${}_{0.5}$Hf${}_{0.5}$NiSn accidentally meets almost perfectly the stable composition and therefore shows the best thermoelectric properties in this series.

The Ti${}_{\left(1-x\right)}$Zr${}_{x}$NiSn system also exhibits three stable phases. Two stable compositions were accidentally synthesized and exhibit the highest values for the thermal conductivity, which can be explained by the better crystallinity. One interesting result in this system is the metallic behavior of the Ti${}_{0.3}$Zr${}_{0.7}$NiSn sample, which is one of the stable compositions. Another result is that the two samples containing two Heusler phases exhibit low values for the thermal conductivity and hence the best thermoelectric properties. The sample Ti${}_{0.6}$Zr${}_{0.4}$NiSn, which consists of one Heusler phase and the binary phase ZrSn${}_{2}$, shows the maximum value for the thermoelectric figure of merit ZT= 1.1 due to the especially low thermal conductivity.

The Ti${}_{\left(1-x\right)}$Hf${}_{x}$NiSn system also shows three stable Heusler phases. The samples, which almost consist of one Heusler phase, show the highest values for the Seebeck coefficient and the highest thermal conductivity due to the higher crystallinity. The samples with two Heusler phases again exhibit low values for the thermal conductivity, but the Ti${}_{0.4}$Hf${}_{0.6}$NiSn sample, which consists of two Heusler phases, has an unusually high thermal conductivity, showing that not every phase decomposition leads to an improvement of the thermal conductivity.

In the Ti${}_{\left(1-x\right)}$(Zr${}_{0.5}$Hf${}_{0.5}$)${}_{x}$NiSn system, also three stable Heusler phases were obtained. Except one sample, all samples with two Heusler phases exhibit low values for the thermal conductivity. The samples, which consist of almost one Heusler phase, exhibit the highest values of the Seebeck coefficient and the highest values of the resistivity. Table 10 summarizes all the compositions of the stable Heusler phases investigated in this work.

## 4. Materials and Methods

The Ti${}_{x}$Zr${}_{y}$Hf${}_{z}$NiSn samples were prepared by arc melting of stoichiometric amounts of the constituents (Ti 99.95%, Zr 97.5%, Hf 97.5%, Ni 99.99%, Sn 99.99%, from Chempur, Karlsruhe, Germany) in an argon atmosphere of 10 mbar. The samples were remelted several times to increase the homogeneity. The resulting polycrystalline ingots were annealed at about 1223 K in an evacuated quartz tube for 7 days afterwards. The crystal structure of all the samples was analyzed by powder X-ray diffraction at room temperature using a Bruker AXS D8 Advance with Mo $K_{\alpha }$ (λ = 0.7093 Å) radiation in reflection mode. A scanning electron microscope (SEM, Jeol JSM-6400, Tokyo, Japan) equipped with an energy dispersive X-ray spectroscopy (EDX) detection system (EUMEX EDX, Heidenrod, Germany) was used to check the homogeneity and stoichiometry of the samples. The measurements were carried out at a pressure of 3 × 10${}^{-6}$ mbar. An acceleration voltage of 20 kV was applied, and an inspection angle of 35${}^{\circ }$ was set up. For the correction of the quantitative data, the ZAFmethod was applied, which relies on the atomic number (Z), absorption (A) and fluorescence (F) effects. The images were acquired via the digital image processing system (DIPS), and the quantitative chemical analysis was performed with the program WINEDS 4.0. To investigate the thermoelectric properties at high temperature, the ingots were cut into discs and bars. The Seebeck coefficients *S* and electrical conductivity σ were determined simultaneously using an LSR-3 (Linseis, Robbinsville, NJ, USA). The thermal conductivity κ was calculated using the relation $\kappa =C_{p}\alpha \rho$, where $C_{p}$ denotes the specific heat capacity, α the thermal diffusivity and ρ the density. The values α were measured by means of the laser flash method using the Netzsch LFA 457 Instrument, Selb, Germany. The density ρ was calculated from the mass and volume of the cut bars. The heat capacities were estimated by means of the Dulong–Petit law. The uncertainties were 3% for the electrical conductivity and thermal diffusivity and 5% for the Seebeck coefficient, thereby leading to an 11% uncertainty in the figure of merit $ZT=\frac{S^{2}\sigma }{\kappa }T$ by propagation of error. We measured the samples numerous times while they were heated up to 600 ${}^{\circ }$C and cooled and verified that there was no degradation in the various sample properties. The above-mentioned uncertainties are derived from these multiple measurements and are in good agreement with the international round-robin study of the thermoelectric transport properties of an *n*-type half-Heusler compound conducted by eleven laboratories throughout the world [29].

The transport properties at low temperatures were determined by a physical properties measurement system (PPMS, Quantum design, San Jose, CA, USA) using the thermal transport option (TTO) for the thermoelectric properties.

## 5. Conclusions

We studied the phase separation in *n*-type thermoelectric half-Heusler materials, showing that the Ti${}_{x}$Zr${}_{y}$Hf${}_{z}$NiSn system is not a solid solution. After reproducing several previously-studied samples consisting of Ti${}_{x}$Zr${}_{y}$Hf${}_{z}$NiSn, we recognized that most of these samples undergo a phase separation. The intention of our study was to determine the origin of these phase separations, understanding the origin and using this knowledge to design phase-separated composite materials with an enhanced thermoelectric performance.

We found that in every quasi-binary system, e.g., Ti${}_{\left(1-x\right)}$Hf${}_{x}$NiSn, three different compositions are stable Heusler phases. The analogy of the composition of the stable phases of each quasi-binary system we studied is remarkable, but can be explained by possible eutectics or peritectics in the quasi-binary systems. Additionally, this analogy is not that surprising, if the chemical relationship of titanium, zirconium and hafnium is taken into account. We obtained twelve phases in addition to the three parent compounds in the Ti${}_{x}$Zr${}_{y}$Hf${}_{z}$NiSn system and constructed a Gibbs triangle for 950 ${}^{\circ }C$ (see Figure 13). The transport properties of all samples were investigated showing that the pure Heusler phases exhibit relatively large Seebeck coefficients and semiconducting behavior.

A second remarkable result of the transport properties is that the phase separation into two Heusler phases can significantly reduce the thermal conductivity without destroying the electronic properties. It was also shown that phase separations resulting in one Heusler phase and a different binary phase have a detrimental effect on the thermoelectric properties. In general, it is easy to synthesize the stable Heusler phases in the Ti${}_{x}$Zr${}_{y}$Hf${}_{z}$NiSn system, and they exhibit transport properties that are usually expected for samples with high crystallinity and low disorder, also indicated by the relatively high thermal conductivity.

As the next logical step following this investigations, we want to use our detailed knowledge of the phase separation in the *n*-type half Heusler materials to design highly efficient thermoelectric composite materials, e.g., using the three stable phases with the composition Ti${}_{0.21}$Zr${}_{0.40}$Hf${}_{0.39}$NiSn(IV), Ti${}_{0.43}$Zr${}_{0.28}$Hf${}_{0.29}$NiSn(III) and Ti${}_{0.68}$Zr${}_{0.17}$Hf${}_{0.15}$NiSn(I). These results strongly underline the importance of phase separations as a powerful tool for designing highly efficient materials for thermoelectric applications that fulfill the industrial demands for a thermoelectric converter.

## 6. Patents

Two patent applications (DE 102012220306 A1 and US 2014/0127070 A1) resulted from the work reported in this manuscript.

## Figures and Tables

**Table 1:** The phase compositions of the different Zr${}_{\left(1-x\right)}$Hf${}_{x}$NiSn samples and the parent samples.

Weighed Composition	Detected Phases	Ratio	a
Zr${}_{\left(1-x\right)}$Hf${}_{x}$NiSn			(Å)
ZrNiSn	ZrNiSn, Sn	97% ZrNiSn	6.121
Zr${}_{0.95}$Hf${}_{0.05}$NiSn	ZrNiSn, Hf	inclusions	6.114
Zr${}_{0.9}$Hf${}_{0.1}$NiSn	ZrNiSn, Hf	inclusions	6.114
Zr${}_{0.8}$Hf${}_{0.2}$NiSn	Zr${}_{0.85}$Hf${}_{0.15}$NiSn(XII), Sn${}_{3}$Zr${}_{5}$, Hf${}_{2}$Ni${}_{2}$Sn	95% (XII)	6.112
Zr${}_{0.7}$Hf${}_{0.3}$NiSn	Zr${}_{0.55}$Hf${}_{0.45}$NiSn(XIII), Zr${}_{0.75}$Hf${}_{0.25}$NiSn(XII)	$\frac{90\%\;(\mathrm{XIII})}{10\%\;(\mathrm{XII})}$	6.105
Zr${}_{0.6}$Hf${}_{0.4}$NiSn	Zr${}_{0.58}$Hf${}_{0.42}$NiSn(XIII), ZrSn${}_{2}$	95% (XIII)	6.101
Zr${}_{0.5}$Hf${}_{0.5}$NiSn	Zr${}_{0.49}$Hf${}_{0.51}$NiSn(XIII)	95% (XIII)	6.101
Zr${}_{0.4}$Hf${}_{0.6}$NiSn	Zr${}_{0.36}$Hf${}_{0.64}$NiSn(XIV), Ni${}_{3}$Sn${}_{4}$	90% (XIV)	6.098
Zr${}_{0.3}$Hf${}_{0.7}$NiSn	Zr${}_{0.29}$Hf${}_{0.71}$NiSn(XIV), ZrSn${}_{2}$	95% (XIV)	6.094
Zr${}_{0.2}$Hf${}_{0.8}$NiSn	HfNiSn, Zr${}_{5}$Sn${}_{3}$	90% HfNiSn	6.092
Zr${}_{0.1}$Hf${}_{0.9}$NiSn	HfNiSn, Zr	inclusions	6.091
Zr${}_{0.05}$Hf${}_{0.95}$NiSn	HfNiSn, Zr	inclusions	6.091
HfNiSn	HfNiSn	97% HfNiSn	6.091

**Table 2:** Compositions of the stable Heusler phases with an error range of 3%.

Stable Compositions
ZrNiSn
Zr${}_{0.78}$Hf${}_{0.22}$NiSn(XII)
Zr${}_{0.55}$Hf${}_{0.45}$NiSn(XIII)
Zr${}_{0.36}$Hf${}_{0.64}$NiSn(XIV)
HfNiSn

**Table 3:** The phase compositions of the different Ti${}_{\left(1-x\right)}$Zr${}_{x}$NiSn samples and the parent samples.

Weighed Composition	Detected Phases	Ratio	a
Ti${}_{\left(1-x\right)}$Zr${}_{x}$NiSn			(Å)
TiNiSn	TiNiSn, Ni${}_{2}$TiSn, Ti${}_{6}$Sn${}_{5}$, Sn	85% TiNiSn	5.939
Ti${}_{0.95}$Zr${}_{0.05}$NiSn	TiNiSn, Ni${}_{2}$TiSn, Ti${}_{6}$Sn${}_{5}$, Zr	85% TiNiSn	5.961
Ti${}_{0.9}$Zr${}_{0.1}$NiSn	TiNiSn, Ni${}_{2}$TiSn, Zr	90% TiNiSn	5.960
Ti${}_{0.8}$Zr${}_{0.2}$NiSn	Ti${}_{0.80}$Zr${}_{0.19}$NiSn(IX), Ti${}_{6}$Sn${}_{5}$	95% (IX)	5.966
Ti${}_{0.7}$Zr${}_{0.3}$NiSn	Ti${}_{0.78}$Zr${}_{0.22}$NiSn(IX), Ni${}_{3}$Sn${}_{4}$	60% (IX)	6.001
Ti${}_{0.6}$Zr${}_{0.4}$NiSn	Ti${}_{0.62}$Zr${}_{0.38}$NiSn(X), ZrSn${}_{2}$	90% (X)	6.086
Ti${}_{0.5}$Zr${}_{0.5}$NiSn	Ti${}_{0.74}$Zr${}_{0.26}$NiSn(IX), Ti${}_{0.36}$Zr${}_{0.64}$NiSn(XI)	$\frac{50\%\;(\mathrm{IX})}{50\%\;(\mathrm{XI})}$	6.005/6.067
Ti${}_{0.4}$Zr${}_{0.6}$NiSn	Ti${}_{0.66}$Zr${}_{0.34}$NiSn(X), Ti${}_{0.23}$Zr${}_{0.77}$NiSn(XI)	$\frac{40\%\;(X)}{60\%\;(\mathrm{XI})}$	6.075
Ti${}_{0.3}$Zr${}_{0.7}$NiSn	Ti${}_{0.29}$Zr${}_{0.71}$NiSn(XI)	97% (XI)	6.204
Ti${}_{0.2}$Zr${}_{0.8}$NiSn	ZrNiSn, Ti${}_{6}$Sn${}_{5}$, Zr	90% ZrNiSn	6.101
Ti${}_{0.1}$Zr${}_{0.9}$NiSn	ZrNiSn,Ti${}_{6}$Sn${}_{5}$	95% ZrNiSn	6.101
Ti${}_{0.05}$Zr${}_{0.95}$NiSn	ZrNiSn, Ti	95% ZrNiSn	6.108
ZrNiSn	ZrNiSn, Sn	97% ZrNiSn	6.121

**Table 4:** Compositions of the stable Heusler phases with an error range of 3%.

Stable Compositions
TiNiSn
Ti${}_{0.80}$Zr${}_{0.19}$NiSn(IX)
Ti${}_{0.66}$Zr${}_{0.34}$NiSn(X)
Ti${}_{0.29}$Zr${}_{0.71}$NiSn(XI)
ZrNiSn

**Table 5:** The phase compositions of the different Ti${}_{\left(1-x\right)}$Hf${}_{x}$NiSn samples and the parent samples.

Weighed Composition	Detected Phases	Ratio	a
Ti${}_{\left(1-x\right)}$Hf${}_{x}$NiSn			(Å)
TiNiSn	TiNiSn, Ni${}_{2}$TiSn, Ti${}_{6}$Sn${}_{5}$, Sn	85% TiNiSn	5.939
Ti${}_{0.95}$Hf${}_{0.05}$NiSn	TiNiSn, Ni${}_{2}$TiSn, Ti${}_{6}$Sn${}_{5}$, Hf	85% TiNiSn	5.940
Ti${}_{0.9}$Hf${}_{0.1}$NiSn	Ti${}_{0.87}$Hf${}_{0.13}$NiSn(VII), Ti${}_{6}$Sn${}_{5}$	90% (VII)	5.955
Ti${}_{0.8}$Hf${}_{0.2}$NiSn	Ti${}_{0.83}$Hf${}_{0.17}$NiSn(VII)	97% (VII)	5.971
Ti${}_{0.7}$Hf${}_{0.3}$NiSn	Ti${}_{0.87}$Hf${}_{0.13}$NiSn(VII), Ti${}_{0.61}$Hf${}_{0.39}$NiSn(VI)	$\frac{50\%\;(\mathrm{VII})}{50\%\;((\mathrm{VI})}$	5.954/5.991
Ti${}_{0.6}$Hf${}_{0.4}$NiSn	Ti${}_{0.64}$Hf${}_{0.36}$NiSn(VI), Ti${}_{6}$Sn${}_{5}$	95% (VI)	5.995
Ti${}_{0.5}$Hf${}_{0.5}$NiSn	Ti${}_{0.59}$Hf${}_{0.41}$NiSn(VI), Ti${}_{0.26}$Hf${}_{0.74}$NiSn(V)	$\frac{30\%\;(\mathrm{VI})}{70\%\;(V)}$	6.038
Ti${}_{0.4}$Hf${}_{0.6}$NiSn	Ti${}_{0.79}$Hf${}_{0.21}$NiSn(VII), Ti${}_{0.27}$Hf${}_{0.73}$NiSn(V)	$\frac{30\%\;(\mathrm{VII})}{70\%\;(V)}$	6.043
Ti${}_{0.3}$Hf${}_{0.7}$NiSn	Ti${}_{0.19}$Hf${}_{0.81}$NiSn(V), Ti${}_{6}$Sn${}_{5}$	85% (V)	6.045
Ti${}_{0.2}$Hf${}_{0.8}$NiSn	Ti${}_{0.19}$Hf${}_{0.81}$NiSn(V), Ti${}_{6}$Sn${}_{5}$	95% (V)	6.071
Ti${}_{0.1}$Hf${}_{0.9}$NiSn	HfNiSn,Ti${}_{6}$Sn${}_{5}$	90% HfNiSn	6.090
Ti${}_{0.05}$Hf${}_{0.95}$NiSn	HfNiSn, Ti${}_{6}$Sn${}_{5}$	95% HfNiSn	6.089
HfNiSn	HfNiSn, Sn	97% HfNiSn	6.091

**Table 6:** Compositions of the stable Heusler phases with an error range of 3%.

Stable Compositions
TiNiSn
Ti${}_{0.83}$Hf${}_{0.17}$NiSn(VII)
Ti${}_{0.64}$Hf${}_{0.36}$NiSn(VI)
Ti${}_{0.24}$Hf${}_{0.76}$NiSn(V)
HfNiSn

**Table 7:** The phase compositions of the different Ti${}_{\left(1-x\right)}$(Zr${}_{0.5}$Hf${}_{0.5}$)${}_{x}$NiSn samples and the parent samples.

Weighed Composition	Detected Phases	Ratio	a
Ti${}_{\left(1-x\right)}$(Zr${}_{0.5}$Hf${}_{0.5}$)${}_{x}$NiSn			(Å)
Zr${}_{0.5}$Hf${}_{0.5}$NiSn	Zr${}_{0.49}$Hf${}_{0.51}$NiSn(XIII)	95% (XIII)	6.101
Ti${}_{0.05}$(Zr${}_{0.5}$Hf${}_{0.5}$)${}_{0.95}$NiSn	Zr${}_{0.53}$Hf${}_{0.47}$NiSn(XIII), Ti${}_{6}$Sn${}_{5}$	90% (XIII)	6.109
Ti${}_{0.1}$(Zr${}_{0.5}$Hf${}_{0.5}$)${}_{0.9}$NiSn	Zr${}_{0.53}$Hf${}_{0.47}$NiSn(XIII), Ti${}_{6}$Sn${}_{5}$	70% (XIII)	6.117
Ti${}_{0.2}$(Zr${}_{0.5}$Hf${}_{0.5}$)${}_{0.8}$NiSn	Ti${}_{0.18}$Zr${}_{0.42}$Hf${}_{0.40}$NiSn(IV), Ti${}_{0.44}$Zr${}_{0.26}$Hf${}_{0.28}$NiSn(III)	$\frac{95\%\;(\mathrm{IV})}{5\%\;(\mathrm{III})}$	6.090
Ti${}_{0.3}$(Zr${}_{0.5}$Hf${}_{0.5}$)${}_{0.7}$NiSn	Ti${}_{0.24}$Zr${}_{0.39}$Hf${}_{0.37}$NiSn(IV), Ti${}_{0.44}$Zr${}_{0.27}$Hf${}_{0.29}$NiSn(III), Ti${}_{6}$Sn${}_{5}$, Sn	$\frac{80\%\;(\mathrm{IV})}{20\%\;(\mathrm{III})}$	6.092/6.013
Ti${}_{0.4}$(Zr${}_{0.5}$Hf${}_{0.5}$)${}_{0.6}$NiSn	Ti${}_{0.30}$Zr${}_{0.35}$Hf${}_{0.35}$NiSn(III), Ti${}_{0.65}$Zr${}_{0.22}$Hf${}_{0.13}$NiSn(I)	$\frac{60\%\;(\mathrm{III})}{40\%\;(I)}$	6.020
Ti${}_{0.5}$(Zr${}_{0.5}$Hf${}_{0.5}$)${}_{0.5}$NiSn	Ti${}_{0.39}$Zr${}_{0.28}$Hf${}_{0.34}$NiSn(III), Ti${}_{0.76}$Zr${}_{0.15}$Hf${}_{0.09}$NiSn(I)	$\frac{50\%\;(\mathrm{III})}{50\%\;(I)}$	6.050/5.986
Ti${}_{0.6}$(Zr${}_{0.5}$Hf${}_{0.5}$)${}_{0.4}$NiSn	Ti${}_{0.46}$Zr${}_{0.26}$Hf${}_{0.27}$NiSn(III), Ti${}_{0.78}$Zr${}_{0.14}$Hf${}_{0.08}$NiSn(I)	$\frac{50\%\;(\mathrm{III})}{50\%\;(I)}$	6.045/5.983
Ti${}_{0.7}$(Zr${}_{0.5}$Hf${}_{0.5}$)${}_{0.3}$NiSn	Ti${}_{0.54}$Zr${}_{0.21}$Hf${}_{0.26}$NiSn(III), Ti${}_{0.74}$Zr${}_{0.14}$Hf${}_{0.11}$NiSn(I)	$\frac{30\%\;(\mathrm{III})}{70\%\;(I)}$	6.001
Ti${}_{0.8}$(Zr${}_{0.5}$Hf${}_{0.5}$)${}_{0.2}$NiSn	Ti${}_{0.89}$Zr${}_{0.11}$NiSn(IX), Ti${}_{0.69}$Zr${}_{0.13}$Hf${}_{0.19}$NiSn(I)	$\frac{60\%\;(\mathrm{IX})}{40\%\;(I)}$	5.973
Ti${}_{0.9}$(Zr${}_{0.5}$Hf${}_{0.5}$)${}_{0.1}$NiSn	Ti${}_{0.87}$Hf${}_{0.13}$NiSn(VII), Ti${}_{6}$Sn${}_{5}$, ZrSn${}_{2}$	70% (VII)	5.957
TiNiSn	TiNiSn, Ni${}_{2}$TiSn, Ti${}_{6}$Sn${}_{5}$, Sn	85% TiNiSn	5.939

**Table 8:** Compositions of the stable Heusler phases in an error range of 3%.

Stable Compositions
Zr${}_{0.55}$Hf${}_{0.45}$NiSn(XIII)
Ti${}_{0.18}$Zr${}_{0.42}$Hf${}_{0.40}$NiSn(IV)
Ti${}_{0.43}$Zr${}_{0.28}$Hf${}_{0.29}$NiSn(III)
Ti${}_{0.67}$Zr${}_{0.18}$Hf${}_{0.13}$NiSn(I)
Ti${}_{0.81}$Zr${}_{0.19}$NiSn(IX)
Ti${}_{0.83}$Hf${}_{0.17}$NiSn(VII)
TiNiSn

**Table 9:** The phase compositions of the different Ti${}_{x}$Zr${}_{y}$Hf${}_{z}$NiSn samples.

Weighed Composition	Detected Phases	Ratio
Ti${}_{x}$Zr${}_{y}$Hf${}_{z}$NiSn		
Ti${}_{0.4}$Zr${}_{0.48}$Hf${}_{0.12}$NiSn	Ti${}_{0.64}$Zr${}_{0.36}$NiSn(X), Ti${}_{0.29}$Zr${}_{0.71}$NiSn(XI), Hf	$\frac{20\%\;(X)}{80\%\;(\mathrm{XI})}$
Ti${}_{0.5}$Zr${}_{0.4}$Hf${}_{0.1}$NiSn	Ti${}_{0.70}$Zr${}_{0.30}$NiSn(X), Ti${}_{0.29}$Zr${}_{0.71}$NiSn(XI), Hf	$\frac{30\%\;(X)}{70\%\;(\mathrm{XI})}$
Ti${}_{0.6}$Zr${}_{0.32}$Hf${}_{0.08}$NiSn	Ti${}_{0.77}$Zr${}_{0.23}$NiSn(IX), Ti${}_{0.39}$Zr${}_{0.61}$NiSn(XI), Hf	$\frac{40\%\;(\mathrm{IX})}{60\%\;(\mathrm{XI})}$
Ti${}_{0.7}$Zr${}_{0.24}$Hf${}_{0.06}$NiSn	Ti${}_{0.83}$Zr${}_{0.16}$NiSn(IX), Ti${}_{0.65}$Zr${}_{0.35}$NiSn(X), Hf	$\frac{50\%\;(\mathrm{IX})}{50\%\;(X)}$
Ti${}_{0.4}$Zr${}_{0.24}$Hf${}_{0.36}$NiSn	Ti${}_{0.70}$Zr${}_{0.16}$Hf${}_{0.14}$NiSn(I), Zr${}_{0.39}$Hf${}_{0.61}$NiSn(XIV)	$\frac{30\%\;(I)}{70\%\;(\mathrm{XIV})}$
Ti${}_{0.5}$Zr${}_{0.2}$Hf${}_{0.3}$NiSn	TiNiSn, Zr${}_{0.38}$Hf${}_{0.62}$NiSn(XIV)	70% (XIV)
Ti${}_{0.6}$Zr${}_{0.16}$Hf${}_{0.24}$NiSn	TiNiSn, Zr${}_{0.40}$Hf${}_{0.60}$NiSn(XIV)	80% (XIV)
Ti${}_{0.7}$Zr${}_{0.12}$Hf${}_{0.18}$NiSn	TiNiSn, Ti${}_{0.66}$Hf${}_{0.34}$NiSn(VI), Zr	70% (VI)
Ti${}_{0.21}$Zr${}_{0.40}$Hf${}_{0.39}$NiSn	Ti${}_{0.21}$Zr${}_{0.40}$Hf${}_{0.39}$NiSn(IV)	97% (IV)
Ti${}_{0.43}$Zr${}_{0.28}$Hf${}_{0.29}$NiSn	Ti${}_{0.43}$Zr${}_{0.28}$Hf${}_{0.29}$NiSn(III)	95% (III)
Ti${}_{0.68}$Zr${}_{0.17}$Hf${}_{0.15}$NiSn	Ti${}_{0.68}$Zr${}_{0.17}$Hf${}_{0.15}$NiSn(I)	95% (I)

**Table 10:** Compositions of the stable Heusler phases with an error range of 3%.

Stable Compositions
TiNiSn
ZrNiSn
HfNiSn
Zr${}_{0.78}$Hf${}_{0.22}$NiSn(XII)
Zr${}_{0.55}$Hf${}_{0.45}$NiSn(XIII)
Zr${}_{0.36}$Hf${}_{0.64}$NiSn(XIV)
Ti${}_{0.80}$Zr${}_{0.19}$NiSn(IX)
Ti${}_{0.66}$Zr${}_{0.34}$NiSn(X)
Ti${}_{0.29}$Zr${}_{0.71}$NiSn(XI)
Ti${}_{0.83}$Hf${}_{0.17}$NiSn(VII)
Ti${}_{0.64}$Hf${}_{0.36}$NiSn(VI)
Ti${}_{0.24}$Hf${}_{0.76}$NiSn(V)
Ti${}_{0.21}$Zr${}_{0.40}$Hf${}_{0.39}$NiSn(IV)
Ti${}_{0.43}$Zr${}_{0.28}$Hf${}_{0.29}$NiSn(III)
Ti${}_{0.68}$Zr${}_{0.17}$Hf${}_{0.15}$NiSn(I)
